# Brazilian Propolis: A Natural Product That Improved the Fungicidal Activity by Blood Phagocytes

**DOI:** 10.1155/2013/541018

**Published:** 2012-12-27

**Authors:** Muryllo Mendes Possamai, Adenilda Cristina Honorio-França, Ana Paula Barcelos Reinaque, Eduardo Luzia França, Paula Cristina de Souza Souto

**Affiliations:** Materials Science Postgraduate Program, Araguaia University Campus, Federal University of Mato Grosso, BR-070, Km 5, 78600-000 Barra do Garças, MT, Brazil

## Abstract

Natural product incorporation into microcarriers increases the bioavailability of these compounds, consequently improving their therapeutic properties. Natural products, particularly those from bees such as propolis, are widely used in popular medicine. Propolis is a powerful treatment for several diseases. In this context, the present study evaluated the effect of propolis *Scaptotrigona* sp. and its fractions, alone or adsorbed to polyethylene glycol (PEG) microspheres, on the activity of human phagocytes against *Candida albicans*. The results show that propolis exerts a stimulatory effect on these cells to assist in combating the fungus, especially as the crude extract is compared with the fractions. However, when incorporated into microspheres, these properties were significantly potentiated. These results suggest that propolis adsorbed onto PEG microspheres has immunostimulatory effects on phagocytes in human blood. Therefore, propolis may potentially be an additional natural product that can be used for a variety of therapies.

## 1. Introduction

The current focus on natural products and alternative medicines has renewed interest in bee products such as honey, royal jelly, pollen, and propolis [1, 2]. Propolis is a sticky dark-coloured material collected by worker bees from the leaf buds or exuded from numerous tree species. Once collected, this material is enriched with salivary and enzymatic secretions and used in the construction, adaptation, and protection of their nests [3, 4]. In this way propolis chemical composition is a direct reflex of the vegetable flora and bee species [5, 6].

In Brazil, besides wide variety of flora there are also several bees species, among which stand out those belonging to Apidae family and Meliponinae subfamily, better known as indigenous stingless bees, and which produce propolis from the resinous material of plants with wax and soil. In this group are found bees of the *Scaptotrigona* genus with twenty four species described and eight of which already identified in Brazil [4, 7–9].

In particular, the propolis shows potential because of its therapeutic properties and possible applications in the pharmaceutical industry [10–12]. A range of biological activities have been attributed to propolis, including immunomodulatory [13, 14], antibacterial [15], fungicidal [16, 17], anti-inflammatory, healing [18], anesthetic [19], and anticarcinogenic effects [20].

On the other hand, many natural products are not therapeutically effective when used without modification. In many cases, this failure is attributed to low concentrations at the therapeutic targets. One factor that influences the bioavailability of natural products is the extensive metabolism that they undergo in vivo by commensals or probiotics during their passage through the intestine and liver, significantly changing the exact species that is found in systemic circulation [21]. Susceptibility to chemical hydrolysis at physiological pH has been demonstrated for natural substances with high biological activity [22].

One alternative to these problems is the use of polymeric microparticle systems, which have shown promise for the adsorption of phytopharmaceuticals. These systems also promote the controlled release of drugs or biologically active hydrophilic or hydrophobic substances [23, 24]. Among the polymers used in the preparation of microcarriers, polyethylene glycol (PEG) has excellent properties such as solubility in both water and organic solvents and the absence of toxicity and antigenicity, which are essential for biomedical applications. There have been multiple studies of the effect of pairing natural or synthetic drugs with PEG microparticle systems [25–33]. These combinations present numerous advantages, such as prolonging residence in the body, decreasing metabolic degradation by enzymes, and reducing or eliminating the immunogenicity of proteins [27]. It is likely that the development of drugs that incorporate natural materials will be able to reduce side effects, decrease costs, and maximize the benefits of natural product formulations to avoid the aforementioned problems.

In this context, the aim of this study was to evaluate the immunomodulatory and fungicidal effects of propolis adsorbed to PEG microspheres on human phagocytes in the blood.

## 2. Materials and Methods

### 2.1. Propolis

Propolis samples were collected in February 2011, directly from beehives of *Scaptotrigona* sp. in a meliponary of Barra do Garças city (15°52′19.4′′S and 52°10′27.03′′ W), eastern region of Mato Grosso state, Brazil, where predominates the Cerrado stricto sensu. The mean annual temperature is 25.5°C, with two well-defined seasons: rainy (October to April) and dry (May to September) seasons. The ratio of annual rainfall is 1750 mm [34].

Propolis samples had resinous aspect, balsamic, with dark brown color, typical vegetable odor, and solid impurities free. After collecting was promptly stored in lidded container for transport.

### 2.2. Preparation of Crude Extract from Propolis

The production of an ethanol extract of propolis was adapted from Miorin [4], where 30 grams of propolis, fragmented into small pieces, was placed in a container with a lid, and 100 mL of absolute ethyl alcohol was added. The solution was left for 7 days at room temperature with periodic agitation. Subsequently, the solution was filtered through Whatman no. 3 filter paper and placed in petri dishes (preweighed). An oven 40°C was used to evaporate the ethanol, resulting in 19.3 g of crude extract with a dark brown, molasses-like appearance.

### 2.3. Chemical Screening of Crude Extract of Propolis

Qualitative chemical screening of hydroalcoholic extract of propolis was performed to verify the presence of cyanogenic glycosides, phenols, tannins, anthocyanidins, anthocyanins, flavonoids, leucoanthocyanidins, catechins, flavanones, flavonols, xanthones, steroids, triterpenoids, saponins, and alkaloids according to Harbone [35]. The following reagents and chemicals were used: alkaloids with Dragendorff's reagent, flavonoids with metallic magnesium and HCl, saponins with the ability to produce foam, reducing sugars with Fehling's reagent, glycosides with Liebermann's test, tannins with ferric chloride, and polysaccharides with iodine solution [24].

### 2.4. Fractionation of Extract of Propolis

Fractions of the propolis extract were obtained by the modification of the classical liquid chromatography method described by Santos et al. [36]. Silica with a 60~230 mesh was used as the stationary phase and organic solvents (hexane, dichloromethane, ethyl acetate, and methanol) were used as the mobile phase. The stationary phase of the column was prepared by filling the column with silica suspended in hexane. Next, 5 grams of propolis was dissolved in hexane and added to the stationary phase. Three hundred milliliters of each mobile phase was eluted through the column after the sample was applied, except that 450 mL of methanol was applied as the final column wash. The fractions eluted with hexane, dichloromethane, ethyl acetate, and methanol were pooled based on related spectrophotometric profiles and then placed in a drying oven at 40°C for three days to remove the solvents. After drying, the samples were weighed so that the solutions of known concentrations could be prepared.

### 2.5. Poly(ethylene glycol) (PEG) Microsphere Preparation

Microspheres were produced in accordance with the method described by Scott et al. [32] and modified by Scherer et al. [33]. Briefly, 20 g of PEG 6000 (synth) was suspended in 100 mL of phosphate-buffered saline (PBS) and then mixed (v/v) with a 2% sodium sulfate solution in PBS and incubated at 37°C for 45 minutes. After incubation, the mixture was diluted 3 : 1 in PBS. The formation of microspheres was thermally induced by heating the solution to 95°C for 5 minutes. A solution (v/v) of the crude extract or fractions and microspheres was incubated for 30 minutes at 37°C for adsorption.

Microspheres of PEG with or without the crude extract or fractions adsorbed were stained with a solution of DyLight fluorochrome-488 (10 *μ*g/mL, Pierce) overnight at room temperature in dimethylformamide at a 100 : 1 molar ratio of PEG: DyLight and subsequently analyzed by fluorescence microscopy.

### 2.6. Blood Samples

A sample of 15 mL of blood was collected from 200 clinically healthy male volunteers aged between 18 and 35. All volunteers signed an informed consent form that was approved by the local Ethics Committee before entering the study.

### 2.7. Separation of Blood Cells

Blood samples were collected into heparinized (25 U/mL) tubes. The cells were separated with a Ficoll-Paque gradient (Pharmacia, Uppsala, Sweden) to produce the preparations of mononuclear cells with 98% purity as analyzed by light microscopy. The purified macrophages were resuspended independently in 199 serum-free medium, to a final concentration of 2 × 10^6^ cells/mL.

### 2.8. Culture of *Candida albicans*

The standard *Candida albicans* strain ATCC 10231 was used in the study. Twenty-four hours prior to testing, the fungal samples were suspended in brain heart infusion broth (BHI) and incubated at 37°C for 24 hours. After growth, the fungi were washed two times in PBS, and the concentration was adjusted to 2.0 × 10^7^ yeast cells/mL [37].

### 2.9. Determination of Concentration-Response Curve

To determine the concentration-response curve, the effects of doses of 0 *μ*g/mL, 10 *μ*g/mL, 50 *μ*g/mL, and 100 *μ*g/mL of propolis and propolis fractions were determined by the release of superoxide anions by phagocytes as described in Section 2\tmspace +\thinmuskip {.1667em}2.9. All experiments were performed in duplicate or triplicate.

### 2.10. Cytotoxic Analysis

The cytotoxic test was conducted using the acridine orange method [38] as described in Section 2\tmspace +\thinmuskip {.1667em}2.10. In this assay, phagocyte viability was evaluated when treated with propolis or propolis fractions. All experiments were performed in duplicate or triplicate.

### 2.11. Release of Superoxide Anion

One of the methods used for functional evaluation of the phagocytes treated with propolis or propolis fractions, either alone or adsorbed to PEG microspheres, was the analysis of oxidative metabolism as measured by superoxide anion release testing in the presence or absence of *Candida albicans*. The cytochrome C reduction method described by Pick and Mizel [39] and adapted by Honorio-França et al. [40] was utilized. Briefly, mononuclear phagocytes in the presence or absence of the fungus were treated with propolis or propolis fractions, either alone or adsorbed to PEG microspheres, for 30 minutes at 37°C. As a control, PBS-treated mononuclear phagocytes were used. After treatment, the cells were washed and resuspended in PBS containing 2.6 mM CaCl_2_, 2 mM MgCl_2_, and 2 mg/mL cytochrome C. The suspensions (100 *μ*L) were then incubated for 60 minutes at 37°C on culture plates. The reduction of cytochrome C was measured in a microplate spectrophotometer at 550 nm. The superoxide anion concentration was calculated according to the following relationship: O_2_^−^ concentration (nmol) × 100 = optical density/6.3 and the results were expressed as nmol/O_2_^−^. All experiments were performed in duplicate or triplicate.

### 2.12. Phagocytosis and Fungicide Activity

In addition to the method described above, phagocytosis and fungicidal activity tests to evaluate the function of cells treated with propolis or propolis fractions, either alone or adsorbed to PEG microspheres, were performed. The acridine orange method proposed by Bellinati-Pires et al. [38] was used for this purpose. Briefly, equal volumes of mononuclear phagocytes and fungal suspensions were treated with propolis or propolis fractions, either alone or adsorbed to PEG microspheres, and incubated for 30 minutes at 37°C with continuous shaking. PBS-treated mononuclear phagocytes and the fungal suspension were used as the control. After incubation, phagocytosis was stopped by incubation on ice. To remove the extracellular fungal cells, the suspensions were washed and centrifuged twice (160 ×g, 10 min, 4°C). Then, the cells were resuspended in serum-free medium 199 and centrifuged. The supernatant was discarded and the pellet was stained with 200 *μ*L of acridine orange (14.4 g/L of PBS) for 2 minutes. The pellet was then resuspended in cold culture 199 medium, washed twice, and observed under a fluorescence microscope at 400x and 1000x magnification. The phagocytosis index was calculated by counting the number of cells ingesting at least 3 yeast cells within a pool of 100 cells. To determine the fungicide ratio, 100 phagocytes that had ingested yeast cells were counted, taking into account the number of live yeast (green) and dead yeast (orange) × 100 [41]. All experiments were performed in duplicate or triplicate.

### 2.13. Statistical Analysis

Analysis of variance (ANOVA) was used to evaluate the viability, superoxide release, phagocytosis, and the phagocytes' fungicide activity. Statistical significance was defined by a *P* value lower than 0.05 (*P* < 0.05).

## 3. Results

### 3.1. Chemical Screening of Crude Extract from Propolis

Chemical screening showed the presence of tannins, phenols, flavones, flavonoids, and xanthones. The chemical composition of the mixture also contained to a lesser degree, flavanones, and resins (Table 1).

### 3.2. Characterization of PEG Microspheres

**Figure 1:**
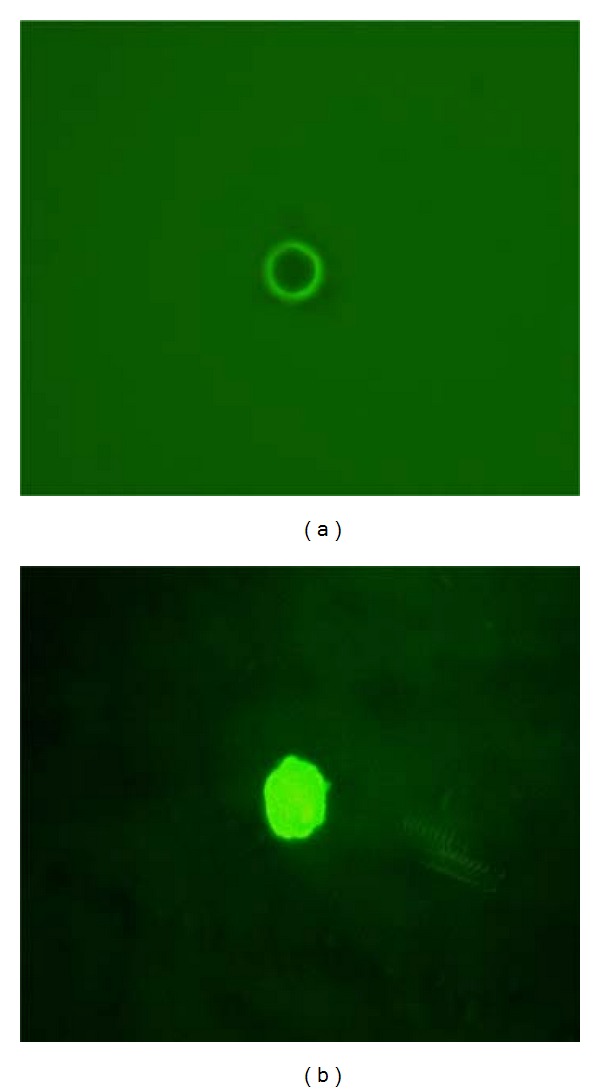
(a) Polyethylene glycol microspheres with a homogeneous surface. (b) Polyethylene glycol microsphere with adsorbed propolis. Heterogeneous adsorption on the microsphere surface.

Fluorescence microscopy was used to analyze the morphology of the PEG microspheres. All PEG microspheres showed similar geometric shapes and sizes with a smooth surface as presented in Figure 1(a).  Figure 1(b) shows the adsorption of propolis in a heterogeneous manner along the microsphere surface. The adsorption is observed as the presence of small regions on the hollow microspheres.

### 3.3. Concentration-Response Curves for Propolis and Propolis Fractions

**Figure 2:**
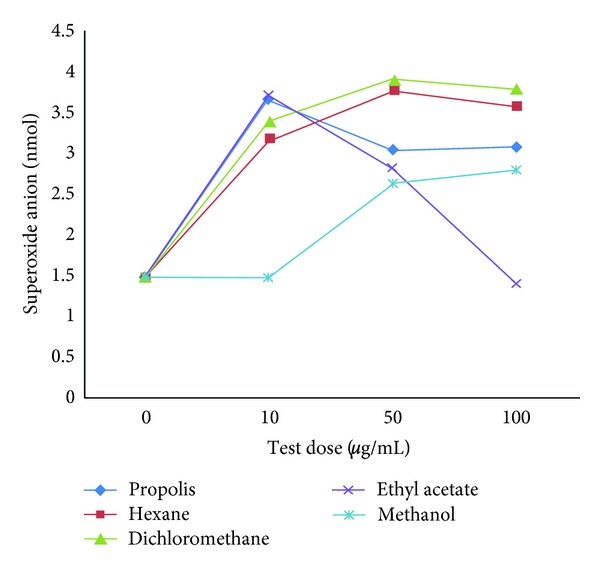
Dose-response curve determined by the superoxide anion release assay for the following dosages of propolis and its fractions: 0 *μ*g/mL, 10 *μ*g/mL, 50 *μ*g/mL, and 100 *μ*g/mL.

To determine the concentration-response relationship, four different doses of propolis and each propolis fraction were examined (0 *μ*g/mL, 10 *μ*g/mL, 50 *μ*g/mL, and 100 *μ*g/mL). Superoxide release by phagocytes upon exposure to propolis and propolis fractions was evaluated, and a correlation between concentration and response was observed, with superoxide release increasing with the size of the dose. Based on the results, we used a test concentration of 50 *μ*g/mL (Figure 2).

### 3.4. Cytotoxic Effect of Propolis and Propolis Fractions on Mononuclear Phagocytes

Neither the PEG microspheres nor propolis affected the viability of the phagocytes. The dichloromethane fraction, when incubated with phagocytes, had a viability index of less than 90% and was not tested in any further assays of functional cell activity. The other fractions did not affect cell viability and were tested for functional activation (Table 2).

### 3.5. Effect of Propolis and Propolis Fractions, Alone or Adsorbed to PEG Microspheres, on Superoxide Anion Release

The PEG microspheres did not alter the superoxide release by phagocytes when compared to spontaneous release. In the presence of the fungus, phagocytes showed O_2_-release at rates similar to spontaneous release. The cells treated with propolis or propolis fractions alone also presented rates similar to that of a spontaneous superoxide release.

Phagocytes treated with the propolis or propolis ethyl acetate fraction adsorbed to PEG showed a significant increase in superoxide release, when comparing release in the presence of the fungus to spontaneous superoxide release (*P* < 0.05).

When incubated with propolis adsorbed onto the microspheres, the cells showed an increase in superoxide release compared with phagocytes exposed only to propolis that was not on microspheres. A similar effect was observed when the phagocytes were incubated with the propolis ethyl acetate fraction adsorbed to the microspheres (Table 3).

### 3.6. Effect of Propolis and Propolis Fractions, Alone or Adsorbed to PEG Microspheres, on Phagocytosis

**Figure 3:**
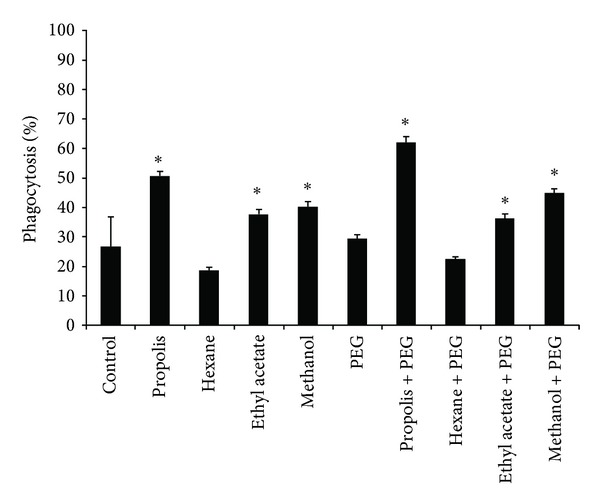
Phagocytic index of phagocytes stimulated with propolis and its fractions, alone or adsorbed to PEG microspheres. (*P < 0.05).

Phagocytes exhibit a basal phagocytic activity in response to *Candida albicans*. This activity did not increase in the presence of PEG microspheres. When phagocytes were stimulated with propolis or propolis fractions, an increase in the phagocytic index was observed, except in the case of the hexane fraction, which significantly reduced the phagocytic activity. The addition of propolis adsorbed onto PEG microspheres resulted in an increased phagocytic activity compared to the treatment with only PEG microspheres or propolis alone (Figure 3).

### 3.7. Effect of Propolis and Propolis Fractions, Alone or Adsorbed to PEG Microspheres, on the Microbicidal Activity of Phagocytes

**Figure 4:**
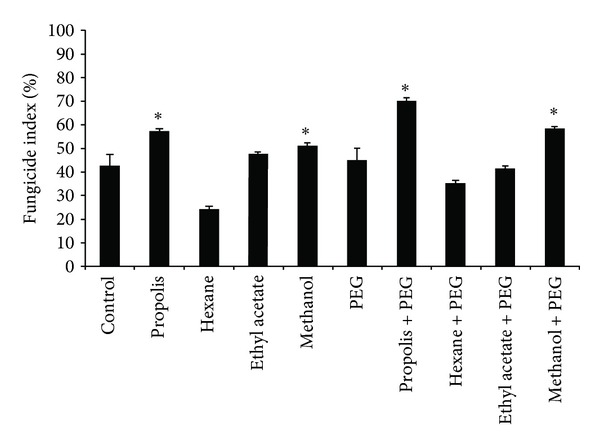
Fungicidal index of phagocytes stimulated with propolis and its fractions, alone or adsorbed to PEG microspheres. (*P < 0.05).

Phagocytes present low fungicidal activity against *Candida albicans*. Increased fungicidal activity was observed when the cells were incubated with propolis and the methanol fraction of propolis. The hexane fraction showed a significant decrease in antifungal activity. An increase in the fungicidal activity was observed when the phagocytes were incubated with PEG microspheres alone (*P* < 0.05, Figure 4).

## 4. Discussion

In the present study, we determined the effect of propolis and the propolis fractions obtained by a polarity gradient and adsorbed to PEG microspheres on the functional activity of phagocytes from human blood.

PEG microspheres are a type of copolymer used for the clinical administration of therapeutics because of their capacity to incorporate drugs [42], their ability to increase the duration of drug exposure or the production of other substances such as enzymes [43], and their role as an important signaling vehicle in immunity [44]. 

In this study, analysis by fluorescence microscopy showed the PEG microspheres to be of regular size, be easily separated from a suspension, and to readily adsorb propolis. The characterization of PEG microspheres using flow cytometry has been previously reported and has shown that this polymer is approximately 5.8 *μ*m of diameter [24, 33].

The use of a polymeric microsphere delivery system for natural products has been described in the literature, and this system is a method that may be useful to deliver a variety of medicinal natural products that could provide additional protection against infection [24, 33]. 

The literature describes the importance of natural products in discovering new drugs [45]. One of the numerous medicinal properties of natural products is the ability to modulate the immune system, by either stimulating or suppressing certain immune response events [14, 46–51]. 

Most studies have been based on the action of propolis without determining the scientific basis of their cytotoxic properties. Propolis is a natural product produced by bees from resinous material collected from a variety of plant species, and it is a complex mixture [52]. In this study the main chemical constituents of this propolis showed were tannins, phenols, flavones, flavonoids and xanthones, flavanones, resins, and to a lesser degree, flavanones and resins. Because it is a mixture, the isolation of the active components that cause this effect is a very long and complex process. Alternatively, fractionation performed with solvents of different polarities allows partial purification of the substances in the mixture, and the examination of the fractions obtained via biological assays enables the determination of some of the metabolites that show therapeutic activity [53].

Here, we demonstrated that the dichloromethane fraction is toxic to phagocytes. This may be caused by the presence of toxic metabolite(s) at higher concentrations in this fraction because, according to Cechinel-Filho and Yunes [53], some compounds exhibit cytotoxic effects in high doses. On the other hand, the fractions obtained from elution with hexane, ethyl acetate, and methanol contain important substances that are able to modulate the activity of phagocytes.

The various types of metabolites obtained by the fractionation of propolis by hexane, dichloromethane, ethyl acetate, and methanol are well known [54–56]. The hexane fraction concentrates steroids, terpenes, and acetophenones. The dichloromethane extract contains lignans, flavonoids, desmethoxy sesquiterpenes, triterpenes, and coumarins. Ethyl acetate isolates flavonoids, tannins, xanthones, triterpene acids, saponins, and phenolic compounds and the methanol extract concentrates glycosylated flavonoids, tannins, saponins, and carbohydrates [53].

Mononuclear phagocytes play an important role in host defense. They produce phagocytic NADPH oxidase, which forms superoxide, and this process is necessary to microbicidal activity and for the success of immune and inflammatory reactions [57]. 

During oxidative stress, cells generate high levels of superoxide radicals. Free radical generation has been reported as an important mechanism for body protection from infections, mainly intestinal infections [58].

Phagocytosis is an important defense mechanism, especially for bacterial and fungal infections. The microbicidal activity of phagocytes is mediated by the production of reactive oxygen species (ROS) and the release of lysosomal enzymes [59]. ROSs are highly reactive molecules as a result of their unpaired electrons. These molecules rapidly react with various biomolecules, leading to DNA damage and the deterioration of membranes through lipid peroxidation and eventually causing cell death [60]. The ROS superoxide anion is a major component of this process, as it is a precursor to other oxygen radicals and essential for effective phagocytosis [61]. 

In this study, it was found that the phagocytes exposed to the fungus *Candida albicans* release a superoxide. This release was not modified in the presence of both the fungus and propolis or propolis fractions. In contrast, propolis or the ethyl acetate fraction adsorbed onto PEG microspheres increased a superoxide release by the phagocytes. This increased effect of propolis when adsorbed to PEG microspheres may be associated with possible protective properties of the PEG microsphere against chemical, physical, or biological degradation (enzymatic action, hydrolysis, oxidation, and changes in pH, among others). Several drugs that are associated with PEG and currently on the market, such as interferon alpha (Pegasys, PEG-Intron), growth hormone (Somavert), asparaginase (Oncaspar), and insulin, have prolonged residence time in the plasma relative to the corresponding free drugs, and the association with PEG has been shown to potentiate the pharmacological effects of the drug [62].

The results of this study confirm the importance of the superoxide anion in fungicidal death. The increase in superoxide release in the presence of PEG microspheres with adsorbed propolis affected the phagocytic and microbicidal activity.

We found that mononuclear phagocytes exposed to propolis adsorbed onto PEG microspheres present the highest levels of superoxide release, phagocytosis, and microbicidal activity. These findings indicate that PEG microspheres with adsorbed propolis stimulate the microbicidal activity of phagocytes in the blood. Similar results were shown using other medicinal plants or hormones [24, 33, 63].

Notably, the phagocytes exposed to PEG microspheres adsorbed the ethyl acetate fraction exhibited an increased superoxide release but presented a low microbicidal activity. This result may suggest that the immunosuppressive action of propolis is related to the presence of a molecule in a group of compounds that are normally isolated in the ethyl acetate fraction, such as flavonoids, tannins, xanthones, triterpene acids, saponins, and phenolic compounds.

Evaluating the results of the tests performed with propolis, the unfractionated extract demonstrates a better efficacy than the fractions, suggesting a possible synergistic effect between multiple chemical components, which is not observed to the same extent when the components are separated by fractionation. According to Bussmann et al. [64], the therapeutic success of the mixtures of natural origin may be associated with an intrinsic relationship between the compounds they contain, given that the studies of the activity of individual substances that comprise these mixtures have been shown to be inactive or toxic.

## 5. Conclusions

In conclusion, the results presented here suggest that propolis adsorbed to PEG microspheres has immunostimulatory effects on phagocytes in human blood, and this system might be used for a variety of therapies based on natural products and could reveal an additional mechanism for treating infections.

## Figures and Tables

**Table 1:** Chemical screening for identification and indication of main chemical constituents of crude extract from propolis.

Analysis	Propolis extract
Cyanogenic heterosides	Absent
Phenols and tannins	Present
Anthocyanins and anthocyanidins	Absent
Flavones, flavonols, and xanthones	Present
Chalcones and aurones	Absent
Leucoanthocyanidins	Absent
Catechins	Absent
Flavanones	Present
Flavonols, flavanones, and/or xanthones	Present
Steroids and triterpenoids	Absent
Saponins	Absent
Strong fixed acids	Absent
Resins	Present
Alkaloids	Absent
Quaternary compounds	Absent
Quinones	Absent
Flavonoids aglycones	Absent
Steroid aglycone triterpenoids	Absent

Notes: present, positive reaction; absent, negative reaction.

**Table 2:** Blood mononuclear phagocyte (MN) viability index in the presence of propolis and its fractions. The results represent the means (±SD) of ten experiments with cells from different individuals (ANOVA *P* > 0.05).

Experimental group	MN phagocytes viability (%)
Control	98,2 ± 0,83
Propolis	90,4 ± 1,14
Hexane	94 ± 1,58
Dichloromethane	88,4 ± 1,14
Ethyl acetate	91,6 ± 2,07
Methanol	91 ± 1,58

**Table 3:** Superoxide anion release of phagocytes in the presence or absence of *Candida albicans*, stimulated by propolis and its fractions, alone or adsorbed to PEG. (*P < 0.05).

MN phagocytes	*Candida albicans *	Superoxide anion release (nmol)
Control	Absence	3,67 ± 0,89
	Presence	4,16 ± 2,71
Propolis	Absence	4,12 ± 1,05
	Presence	4,72 ± 2,30
Hexane	Absence	4,21 ± 1,38
	Presence	5,00 ± 2,04
Ethyl acetate	Absence	4,31 ± 0,94
	Presence	4,00 ± 1,46
Methanol	Absence	4,30 ± 0,86
	Presence	3,62 ± 1,58
PEG	Absence	3,30 ± 0,83
	Presence	3,03 ± 0,49
Propolis + PEG	Absence	19,41 ± 0,50*
	Presence	17,44 ± 1,13*
Hexane + PEG	Absence	4,63 ± 1,42
	Presence	4,85 ± 1,63
Ethyl acetate + PEG	Absence	15,77 ± 0,57*
	Presence	15,48 ± 0,60*
Methanol + PEG	Absence	4,51 ± 1,00
	Presence	4,59 ± 1,62
